# Airborne Microplastics in Spanish Schools: Abundance, Morphology, Polymer Composition and Aerodynamic Size in Urban and Peri-Urban Settings

**DOI:** 10.3390/polym18212784

**Published:** 2026-11-07

**Authors:** Eugenia Battellocchi García, María José Gómez Ramos, María Dolores Hernando Guil, Laura Cortés Corrales, Chen Wang, José Luis Oller Serrano, Harshit Sahai, Maria Jesús Martínez Bueno, Amadeo Rodríguez Fernández-Alba

**Affiliations:** 1 Department of Chemistry and Physics, Research Centre for Mediterranean Intensive Agrosystems and Agri-Food Biotechnology (CIAMBITAL), University of Almería, Agri-Food Campus of International Excellence (ceiA3), 04120 Almería, Spain; eugeniabg@ual.es (E.B.G.); mjramos@ual.es (M.J.G.R.); joseluisoller@ual.es (J.L.O.S.); harshitsahai@ual.es (H.S.); mjbueno@ual.es (M.J.M.B.); 2 Experimental Station of Arid Zones, Spanish National Research Council (EEZA-CSIC), Ctra. Sacramento s/n, La Cañada de San Urbano, 04120 Almería, Spain; hernando.dolores@inia.csic.es (M.D.H.G.); lauracortes@ual.es (L.C.C.); 3 School of Environmental and Chemical Engineering, Shanghai University, Shanghai 200444, China; wangchen0227@shu.edu.cn

**Keywords:** airborne microplastics, school environments, polymer characterization, indoor–outdoor, µ-FTIR

## Abstract

Airborne microplastics (MPs) are emerging contaminants of concern in school environments, where children may experience prolonged exposure. This study assessed airborne MPs at three Spanish schools located in contrasting settings: a dense urban area in Madrid (A), a residential area in Almería (B), and an agricultural peri-urban area in Almería surrounded by greenhouses (C). An additional simultaneous indoor–outdoor comparison was performed at School B. Air samples were collected using active samplers with 25 µm stainless-steel meshes, and particles were characterised by stereomicroscopy, µ-FTIR spectroscopy, FESEM, and FTIR. Fibres were the predominant morphology, and polyethylene terephthalate (PET) was the most abundant synthetic polymer. School A showed the highest abundance of synthetic fibres, while polymer composition differed significantly among schools (χ^2^ (12) =41.77, *p* < 0.0001). Indoor samples contained more particles than outdoor samples, although natural-to-synthetic proportions were similar; PP, PA, and PE contributed relatively more indoors, whereas PET predominated outdoors. Only 0.45% and 0.13% of particles fell within the <10 µm and <4 µm estimated aerodynamic-size ranges, respectively, all being fibres. Overall, airborne MP occurrence varied with location and indoor–outdoor setting, while selected PET and PP fibres showed polymer-dependent surface and chemical alteration.

## 1. Introduction

Plastic materials have been integrated into a wide variety of markets and industries due to their desirable characteristics, such as resistance to degradation, malleability, lightness, and low cost, which are key attributes that make plastic a highly versatile and adaptable material [1]. However, various environmental conditions cause these materials to degrade and break down into smaller particles, known as microplastics (MPs) when their size is less than 5 mm [2].

Recently, concern has increased regarding plastic particles that can disperse in the air and be inhaled by humans, potentially causing adverse effects on different systems, such as the respiratory and endocrine systems, as well as other organs [3].

Airborne MPs represent a relatively new phenomenon and an emerging concern. Their small size, varied shapes, diverse composition and wide range of physicochemical properties enable multiple exposure pathways, including inhalation, ingestion and dermal contact. This complexity raises questions about their short- and long-term effects on human health. Additionally, these particles can act as vectors or reservoirs for chemical substances and microorganisms, transporting them deep into the respiratory tract upon inhalation, thereby increasing their potential biological impact [4].

Schools have been identified as key environments for air quality studies, mainly because children spend a large proportion of their time in enclosed classrooms, which are often characterised by limited ventilation, high occupancy density, and frequent particle resuspension [5]. As a result, children may be continuously exposed to complex mixtures of airborne pollutants, making schools highly relevant settings for exposure assessment.

Furthermore, children represent a particularly vulnerable group because, relative to their weight, they have higher breathing rates and therefore inhale a greater volume of air and, consequently, more pollutants than adults [6]. In addition, their higher level of physical activity further increases minute ventilation, that is, the volume of air inhaled per minute [7,8]. Importantly, developing children’s lungs are especially sensitive to the adverse effects of inhaled particles, which may result in more pronounced and long-lasting health impacts [5].

The shape and size of MPs are critical determinants of their aerodynamic behaviour and, consequently, of their transport and deposition within the respiratory tract. Fibres exhibit aerodynamic properties that differ substantially from those of spherical particles, affecting both their inhalation efficiency and deposition pattern. In this context, it is important to distinguish between inhalable, thoracic, and respirable fractions [9]. The inhalable fraction comprises particles that can enter the nose or mouth during breathing, the thoracic fraction includes those capable of penetrating beyond the larynx into the tracheobronchial region, and the respirable fraction refers to particles sufficiently small to reach the alveolar region of the deep lung. Therefore, particle morphology and size distribution are essential parameters for assessing the potential health relevance of airborne MPs [10].

From a respiratory exposure perspective, particle behaviour in air and within the respiratory tract is more appropriately described by aerodynamic diameter (AED) than by geometric size alone. AED is defined as the diameter of a unit-density sphere having the same settling velocity as the particle of interest [9]. AED depends on particle size, density and shape. Consequently, particles with similar geometric dimensions may exhibit different aerodynamic behaviour depending on their physicochemical and morphological properties [11]. Once inhaled, MPs may penetrate different regions of the respiratory tract depending on their aerodynamic properties. In general, larger particles tend to deposit predominantly in the extra thoracic region, thoracic particles may penetrate the tracheobronchial airways, and sufficiently small respirable particles can reach the alveolar region [10]. In particular, MPs with an AED < 10 μm have the potential to penetrate the lower respiratory tract [9].

Beyond their aerodynamic behaviour, the environmental ageing of MPs is another relevant factor influencing their physicochemical properties and potential fragmentation. UV-induced oxidation is one of the most studied mechanisms involved in MP formation and changes in the functional groups of polymers. This process occurs when polymers absorb UV radiation, generating radicals that initiate oxidation chain reactions and lead to the formation of oxygen-containing functional groups, such as carbonyl and hydroxyl groups. Throughout time, UV oxidation causes embrittlement, discoloration and increased fragmentation [12]. Therefore, assessing surface and chemical alterations associated with oxidative ageing can provide additional information on the environmental transformation of airborne MPs.

Although airborne MP pollution is a relatively under-explored field, particularly in school environments, a few methodological approaches have been used to assess its presence. Some studies have quantified MPs through the analysis of settled indoor dust [5], while others have used passive samplers to evaluate atmospheric deposition [13]. Furthermore, investigations of school air quality have examined the influence of ventilation and air purifiers on airborne particle concentrations [7,14].

Additional evidence has been provided by studies examining MPs in educational and other indoor environments. [15] characterized airborne MPs in northern New Jersey, including a university classroom, and reported differences in particle morphology and polymer composition between indoor and outdoor samples. [16] investigated indoor and outdoor PM_10_ at a school in Estarreja, Portugal, finding significantly higher mean MP mass concentrations indoors and seasonal differences between sampling campaigns. More recently, a study conducted in classrooms across five primary schools in Barcelona combined polymer-specific mass measurements with respiratory deposition modelling to assess potential exposure in children [17]. These findings reinforce the relevance of educational environments for MP exposure research and highlight the complementary information provided by particle-resolved characterization, mass-based quantification and deposition measurements.

In this context, the present study was primarily designed to characterize outdoor airborne MP contamination in school environments, with particular emphasis on the temporal and spatial variability of polymer types, particle shape, and size distribution. Measurements were performed at schools located in three contrasting environmental settings to assess the influence of surrounding land use on MP occurrence and characteristics. An indoor–outdoor comparison at one school was additionally included as a complementary evaluation of differences in exposure between both microenvironments.

## 2. Materials and Methods

### 2.1. Sampling Area

Air monitoring was conducted in three schools located in central and southern Spain. The first sampling site was situated in Madrid, the capital of Spain, which is characterised by a continental Mediterranean climate. The school, hereafter referred to as School A, is in a densely populated urban area with moderate to high traffic intensity. Population density around each school was estimated within a 2-km radius (12.56 km^2^) using Horizon Mapper, based on WorldPop 2020 gridded population estimates [18]. The estimated population density around School A was 15,241 inhabitants/km^2^.

The other two sampling sites were in Almería, located in south-eastern Spain with a semi-arid Mediterranean climate, which is considered one of the driest in Europe. School B is located in a predominantly residential urban area with moderate vehicle and pedestrian traffic, with an estimated population density of 4808 inhabitants/km^2^ within the same 2-km radius. In contrast, School C, is situated in an agricultural and peri-urban area near the coast, with an estimated population density of 304 inhabitants/km^2^. The surrounding environment is predominantly characterised by agricultural land use and greenhouse farming, with limited residential development and low population density.

In addition, comparative indoor–outdoor sampling was carried out at School B. Air samples were collected simultaneously inside the classroom and in the school playground, enabling direct comparison under identical environmental conditions.

Outdoor sampling was conducted in paved school playgrounds, using the same sampling area for all campaigns at each school. Five samplers operated simultaneously, with one positioned centrally and the remaining four to the north, south, east and west. The devices were placed directly on the ground with the air inlets facing upwards, at a height of 12 cm above the ground. Pupils were present elsewhere in the playground but remained away from the sampling area. Indoor sampling at School B followed the same five-position arrangement, with the samplers placed on pupils’ desks. The classroom, normally occupied by approximately 25–30 pupils, was sampled immediately after they had left and remained unoccupied during sampling. Doors and windows were closed at the start of sampling and remained closed throughout.

Sampling was carried out under the conditions shown in Table 1. The sampling period was selected to reflect typical school activities, during which human presence and movement can contribute to the emission and resuspension of particles. Conducting the sampling during different months also allowed the assessment of potential variability related to environmental factors, such as temperature and relative humidity, which may influence the suspension, transport, and deposition of airborne particles.

### 2.2. Sample Collection and Extraction

A strict contamination prevention protocol [19] was followed during the sampling and analysis stages in order to minimize cross-contamination by microplastics and ensure the reliability of the results. Prior to each sampling, the stainless-steel collection meshes and all glassware used were cleaned through successive baths of dichloromethane and pre-filtered methanol (both from Honeywell Specialty Chemicals Seelze GmbH, Seelze, Germany),then heat-treated in an oven at 200 °C for 24 h. All laboratory sample-handling procedures were carried out wearing white cotton laboratory coats and nitrile gloves, and under a closed laminar flow hood, with a vertical airflow of 0.6 m/s.

Likewise, the distilled water and all reagents used during the extraction process were previously filtered at least twice using 0.45 µm filters in a vacuum filtration system. Samples and materials were covered with aluminum foil when they were not manipulated to prevent airborne contamination.

In addition, a field blank consisting of a pre-cleaned stainless-steel mesh was included in each sampling campaign. It underwent the same transport and handling procedures as the samples but was not installed in a sampler, allowing possible contamination during transport and handling to be assessed. It was subsequently subjected to the same processing and analytical procedures as the samples. Only cellulose fibres were detected in the field blanks, and their counts were subtracted from the corresponding cellulose counts for each sampling campaign.

Filtration blanks were also prepared by passing distilled water, obtained directly from an in-house laboratory water distillation system, without sample material through cellulose ester membranes of the same type used for sample processing, to assess potential particulate contamination associated with the water and the membranes.

In this study, five active portable air-samplers previously developed to collect airborne MPs [20], were used, including small particles potentially relevant to inhalation exposure within the size range accessible to the analytical workflow. Each air-sampler was equipped with an integrated suction turbine that drew air through a stainless-steel mesh with a nominal aperture of 25 μm, retaining airborne particles on the mesh. No impactor or other aerodynamic size-selection stage was used. An important methodological consideration is that the nominal aperture used in the present study does not represent a sharp aerodynamic size cut-off.

The devices incorporated an integrated flow meter and temperature sensor for real-time monitoring of sampling conditions. A remote-control system connected to a mobile application enabled sampler control and recording of operational parameters, allowing the sampled air volume to be determined. Each sampler operated at a flow rate of 25–30 L/min until 1 m^3^ of air had been collected, resulting in a total sampled air volume of 5 m^3^ per campaign. This sampling volume was selected with the aim of obtaining a representative number of airborne particles present in the environment.

Once the sampling was complete, each stainless-steel mesh was individually transferred to a separate pre-cleaned 250 mL glass jar to prevent external contamination. The samples were then transported to the laboratory for storage and further processing and analysis. The five meshes from each sampling campaign were extracted and analysed separately; neither the meshes nor their extracts were pooled.

In the laboratory, the particles retained on each mesh were recovered by an aqueous extraction process. For this purpose, 75 mL of previously filtered distilled water was added to each glass jar. The sealed jars were then placed in an ultrasonic bath for 15 min to promote the disaggregation and release of the adhered particles from the mesh. The entire contents of each jar, including any settled particles, were vacuum-filtered separately through a cellulose ester filter (S-Pak Filters, pore size 1.2 μm, diameter 47 mm, Merck Millipore, Milford, MA, USA). Each jar was subsequently rinsed with additional previously filtered distilled water to recover particles adhering to the walls, and the rinsate was filtered through the same membrane. The resulting filters were stored in Petri dishes to prevent contamination. All material recovered from each mesh was subjected to the analytical workflow described in Section 2.3 without subsampling.

### 2.3. Analysis, Quantification and Compositional Characterization

After the extraction process, the particles retained on the cellulose ester filters were examined using a Leica S9i stereomicroscope (Leica Microsystems, Wetzlar, Germany) equipped with an integrated digital camera capable of capturing images of up to 10 megapixels.

The observed microparticles were classified according to their morphology and colour. Based on their morphology, and following criteria commonly established in the literature, three main categories were defined: fragments, films, and fibres [1]. In addition, each particle was assigned to a specific colour group, including black/purple, blue, red/pink, green, brown/orange, and translucent. During the microscopic inspection, all white fibres were excluded from the analysis, as they could originate from contamination associated with the laboratory coats used during the handling and extraction of the samples. This dual classification allowed a more detailed description of the diversity of particles present in the samples.

All microparticles suspected to be MPs were individually counted and recorded. This procedure involved visual inspection using a 1x objective lens, which provides a magnification range between 6x and 55x. The images obtained were processed using LAS-X software (version 3.7; Laica Microsystems, Wetzlar, Germany) to determine several relevant morphometric parameters, including length, width, area, roundness, aspect ratio, and curve ratio, among others.

After particle counting and morphological characterization, visually suspected MPs were individually transferred from the cellulose ester filters onto macroporous silicon membrane filters (pitch 12 µm, pore length 500 µm, pore diameter 4–6 µm, MakroPorP12M5-500, macroporous silicon membranes (SmartMembranes GmbH, Halle (saale), Germany) using a fine stainless-steel needle under stereomicroscopic observation. The transfer was performed under dry conditions, without adding any liquid. The silicon membranes were then re-examined under the stereomicroscope to verify the presence and position of each transferred particle, ensuring correspondence between the particles counted and those subsequently analysed. All counted particles were individually analysed using micro-Fourier Transform Infrared spectroscopy (μ-FTIR) with a Nicolet iN10 microscope (Thermo Fisher Scientific, Madison, WI, USA) operating in semi-automated mapping mode procedure in OMNIC Picta software (version 1.9.1.22; Thermo Fisher Scientific, Madison, WI, USA). The operator defined the area of the silicon membrane by selecting its four corners, after which the instrument automatically acquired spectra at positions distributed across the defined area.

Spectra were acquired in transmission mode over a spectral range from 4000 to 650 cm^−1^, using a spectral resolution of 8 cm^−1^ and accumulating 16 scans per spectrum. To enhance signal quality and minimize background noise, the internal mercury cadmium telluride (MCT; Thermo Fisher Scientific, Madison, WI, USA) detector was cooled with liquid nitrogen. In addition, a calibration procedure was performed at the beginning of each analytical session to correct spectral interferences caused by atmospheric carbon dioxide and water vapor, ensuring the reliability of the measurements.

Finally, the obtained spectra were carefully examined and compared with the polymer and additive spectral libraries provided by Thermo Fisher Scientific to determine the chemical composition of the particles. Colour and morphology were used for preliminary screening and morphological characterization, whereas classification as synthetic or non-synthetic material was based on spectroscopic identification. A spectral match higher than 65% was considered indicative of a positive identification of plastic materials [21]. Spectra with match scores below this threshold were discarded to reduce the likelihood of false-positive polymer identification.

This analytical workflow ensured the reliable determination of the morphological properties and the chemical composition of the detected microplastic particles.

### 2.4. Morphometric Characterization and Estimation of an Aerodynamic Diameter Proxy

During visual inspection with the stereomicroscope, particles suspected of being microplastics were identified and photographed for subsequent processing using LAS X 3.7 software. This software allows individual particles to be segmented to obtain contour-based morphometric descriptors and characterize their size and shape.

Among the parameters obtained were the projected area (µm^2^), maximum length (µm), average width (µm) and in the case of fibres, the curve length of the centerline (µm) was also determined, allowing a more accurate description of their morphology. Additionally, further descriptors were extracted to evaluate the shape, elongation, and irregularity degree of the particles. These included the aspect ratio (length/width), the equivalent circular diameter (ECD; µm), defined as the diameter of a circle with the same projected area, and Feret 0 (µm). Additional shape-related parameters were also determined, such as roundness, fullness ratio, shape factor, and curve ratio.

To describe the size of irregular fragments and highly elongated fibres, it was necessary to define a geometric diameter indicator (*dg*) based on the two-dimensional measurements obtained from image analysis. This parameter allows particles with complex geometries to be represented using a single descriptor that can be compared across different morphologies.

For fragments and films, dg was considered equivalent to the equivalent circular diameter obtained from the projected area (*dg* $=\mathrm{ECD}=\sqrt{4A/\pi }$), where A represents the projected particle area. For fibres, since their aerodynamic behaviour is mainly controlled by their transverse dimension (width), but is also influenced by particle thickness and irregularity, *dg* was estimated as a combination of both dimensions, ECD and width. This formulation was intended to reduce the influence of fibre length on the representative size while retaining information from both the projected particle area and its transverse dimension.

The true aerodynamic diameter (*da*) cannot be directly determined from two-dimensional microscopy data because aerodynamic behaviour also depends on three-dimensional particle geometry, orientation and density. Therefore, an estimated aerodynamic diameter proxy indicator (*da,proxy*) was estimated and used for comparative purposes.

Aerodynamic diameter is defined as the diameter of a unit-density sphere (1 g/cm^3^) with the same terminal settling velocity as the considered particle [22]. In this study, *da,proxy* was estimated by considering particle size, the polymer density and a morphology-based shape correction factor derived from image descriptors, according to Equation (1):

(1) $$d_{a,\mathrm{proxy}}=d_{g}\sqrt{\frac{\rho_{p}}{\rho_{0}\cdot \;\chi }}$$

where *dg* is the geometric diameter, ρ_p_ is the reference density of the polymer randomly assigned to each particle, ρ_0_ is the reference density (1 g/cm^3^), and χ is a dimensionless, image-derived shape correction factor reflecting particle elongation, irregularity and deviation from an ideal spherical geometry. Polymer identity was randomly assigned to each particle using probabilities based on the relative abundances of synthetic polymers determined by µ-FTIR. The reference density of the assigned polymer was then used in the calculation. This procedure used the measured polymer proportions but did not preserve the correspondence between each particle’s morphology and its individual polymer identification. The densities applied were PP 0.9 g/cm^3^, PE 0.92 g/cm^3^, PET 1.38 g/cm^3^, PA 1.14 g/cm^3^, PAN 1.18 g/cm^3^, PTFE 2.2 g/cm^3^ and PU 1.2 g/cm^3^.

Since the dynamic shape factor cannot be directly determined from two-dimensional microscopy data, χ was estimated using an empirical approach for comparative purposes. For fragments and films, χ was assigned according to particle roundness, if lower roundness values indicate more angular and irregular morphologies and therefore a greater deviation from a spherical geometry. The following intervals were established: χ = 1.6 for roundness ≥0.75, χ = 2.0 for roundness between 0.55 and 0.75, χ = 2.5 for roundness between 0.35 and 0.55, and χ = 3.0 for roundness <0.35. For fibres, χ was assigned according to aspect ratio, considering that higher values indicate more elongated particles and therefore a greater aerodynamic deviation from a spherical geometry. Values of χ = 1.5, 2.0, 2.5 and 3.0 were assigned for aspect ratio <3, 3–10, 10–20 and >20, respectively. These values were used as empirical correction factors for comparative purposes and were defined specifically for this study. The intervals were established with reference to the observed morphological descriptors, whereas the assigned χ values represent assumed correction factors for exploratory comparisons.

The resulting *d_a_*_,*proxy*_ values should therefore be interpreted as semi-quantitative indicators of relative aerodynamic behaviour rather than as experimentally determined aerodynamic diameters.

### 2.5. FESEM-Based Evaluation of Microplastic Degradation

From the total number of particles detected, a subset of four representative fibres was examined in terms of their physical and surface properties using a field emission scanning electron microscope (FESEM; Carl Zeiss Microscopy GmbH, Sigma 300 VP, Oberkochen, Germany) fitted with a Gemini detection system and an Everhart–Thornley secondary electron detector. Instrument control and image acquisition were carried out using SmartSEM™ software (Carl Zeiss Microscopy GmbH, Jena, Germany). Images were acquired under high-vacuum conditions with the SE2 detector, employing an accelerating voltage (EHT) of 3 kV and a working distance (WD) of 5.0 mm. Before imaging, the samples were fixed onto aluminum stubs with double-sided conductive carbon tape and coated with a thin gold layer (~5 nm) using a sputter coater (LEICA EM ACE 200, Leica Microsystems, Vienna, Austria) to improve surface conductivity.

### 2.6. Statistical Analysis

An exploratory partial least squares discriminant analysis (PLS-DA) was performed to evaluate differences in airborne particle concentration profiles among schools. Concentrations of fibres, fragments and films were expressed as particles per cubic metre/particles/m^3^), considering the total sampled air volume of 5 m^3^ per campaign and are reported as mean values per school (*n* = 3 campaigns each). The predictor matrix X (9 campaigns × 3 variables: fibres, fragments and films) was autoscaled (mean-centred and divided by the standard deviation of each variable) so that all morphologies contributed equally irrespective of their absolute concentrations. School identity was encoded as a binary response matrix Y (9 × 3), with one column per school.

Calculations were performed in Python 3.15.2 with the PLSRegression function of scikit-learn. A model with two latent variables (LV1 and LV2) was retained for exploratory analysis and two-dimensional visualization. Campaign scores on LV1 and LV2 were plotted to visualize their separation according to school, while X-loadings indicated the contribution of each morphological variable to the latent-variable representation.

Model fit was described using R^2^X and R^2^Y. R^2^X was calculated as in Equation (2), where SSE_x_ is the sum of squared residuals after reconstruction of the scaled predictor matrix from the two-component model, and TSS_x_ is the total sum of squares of that matrix.

R^2^Y was calculated as Equation (3), where RSS is the sum of squared differences between observed and fitted binary responses, and TSS is the sum of squared deviations of the observed responses from their column means.

(2) $$R^{2}X=1-\frac{\mathrm{SS}E_{x}}{\mathrm{TS}S_{x}}$$

(3) $$R^{2}Y=1-\frac{\mathrm{RSS}}{\mathrm{TSS}}$$

Predictive performance was assessed using leave-one-out cross-validation (LOO-CV). Each campaign was excluded in turn, and a two-component model was fitted using the remaining eight campaigns to predict the excluded observation. Centring and scaling parameters were estimated exclusively from the training data within each fold. Each excluded campaign was assigned to the school with the highest predicted response value. Classification accuracy was calculated as the proportion of correctly classified campaigns. Q^2^Y was calculated according to Equation (4), where PRESS is the sum of squared differences between observed binary responses and their cross-validated predictions, and TSS is calculated from the observed response matrix around its overall column means.

(4) $$Q^{2}Y=1-\frac{\mathrm{PRESS}}{\mathrm{TSS}}$$

Permutation tests were used to assess whether Q^2^Y and classification accuracy exceeded those expected under random assignment of school labels. Labels were randomly permuted 999 times using a fixed random seed of 42. For each permutation, the two-component model and the complete LOO-CV procedure were repeated. For each metric, the *p*-value was calculated as [5], where b is the number of permuted values equal to or greater than the observed value.

(5) $$p\text{-}\mathrm{value}=\frac{\left(b+1\right)}{\left(999+1\right)}$$

## 3. Results

### 3.1. Abundance and Morphological Characteristics

The abundance and morphological distribution of particles differed among the three sampling locations (Table 2). Fibres were the predominant morphology, accounting for 94.89% of the total particles detected, whereas fragments and films represented only 2.96% and 2.15%, respectively. Fibres were consistently detected across all sampling locations and campaigns. School A showed the highest overall particle abundance, while Schools B and C exhibited lower values. Fragments showed a more heterogeneous distribution, with the highest abundance observed at School C during the first sampling campaign.

Across all sampling locations and morphological categories, particles of natural origin were more abundant than synthetic particles. Among the non-plastic materials identified, cellulose was markedly more abundant than cotton and rayon. These materials were excluded from the calculation of synthetic microplastic concentrations because of their non-plastic nature.

**Figure 1:**
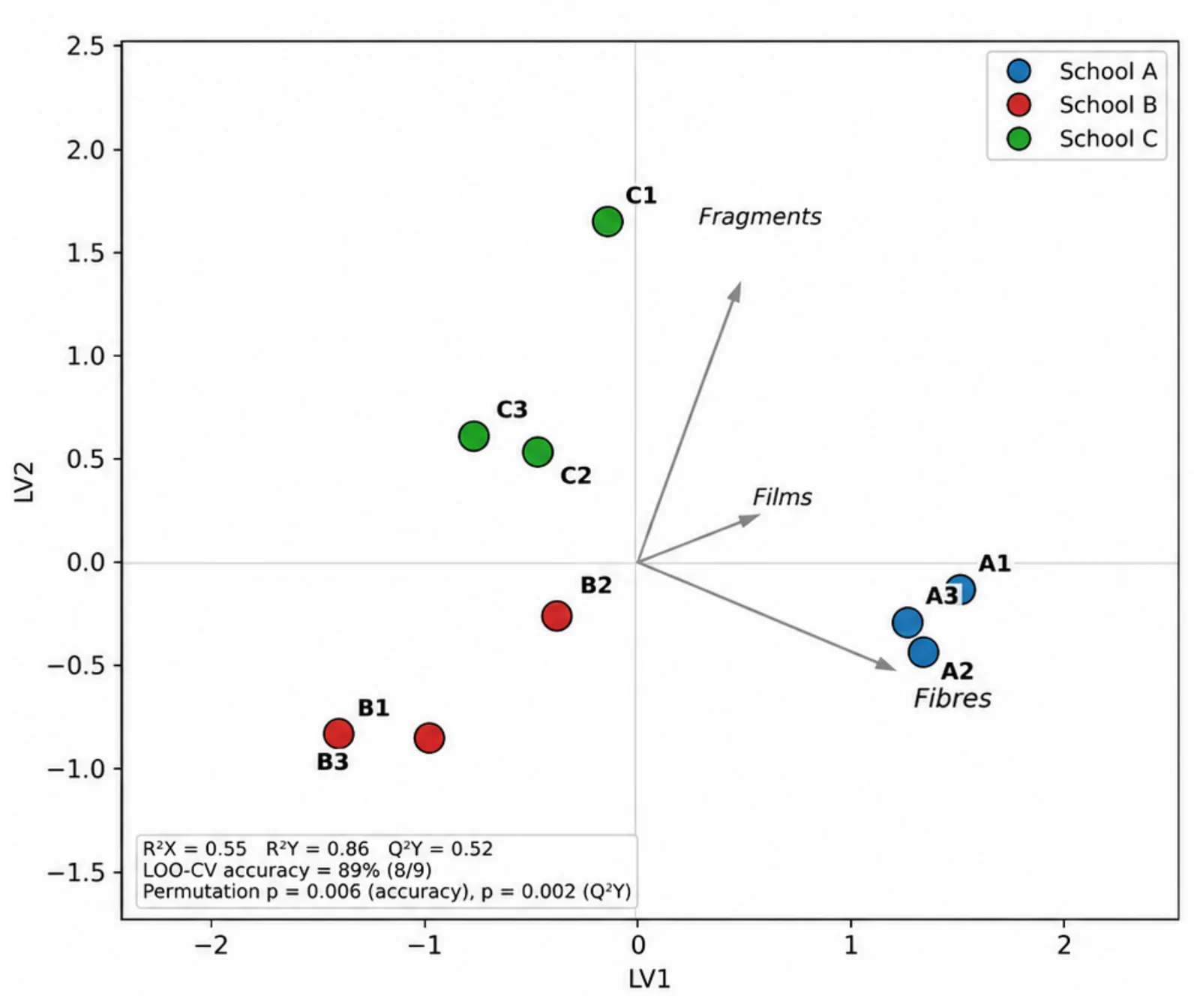
Partial Least Squares Discriminant Analysis (PLS-DA) score plot of particle concentration profiles across the nine sampling campaigns, grouped by school (A, B, C). Vectors represent the loadings of each morphological variable on the first two latent variables (LV1, LV2).

The exploratory two-component PLS-DA model based on total concentrations of fibres, fragments and films, including both plastic and non-plastic particles, explained 63.2% of the variance in X (R^2^X) and 85.8% of the variance in the encoded school response (R^2^Y). Leave-one-out cross-validation yielded a Q^2^Y of 0.516 and a classification accuracy of 88.9% (eight of nine campaigns), with campaign C2 assigned to School B. Permutation tests yielded *p* = 0.006 for classification accuracy and *p* = 0.002 for Q^2^Y. In the score plot (Figure 1), School A campaigns were separated from Schools B and C along LV1, whereas LV2 partially separated School C from School B. Fibres had the highest VIP score (1.23), followed by fragments (1.03) and films (0.65). These findings suggest differences in total airborne particle concentration profiles among the sampled schools.

**Figure 2:**
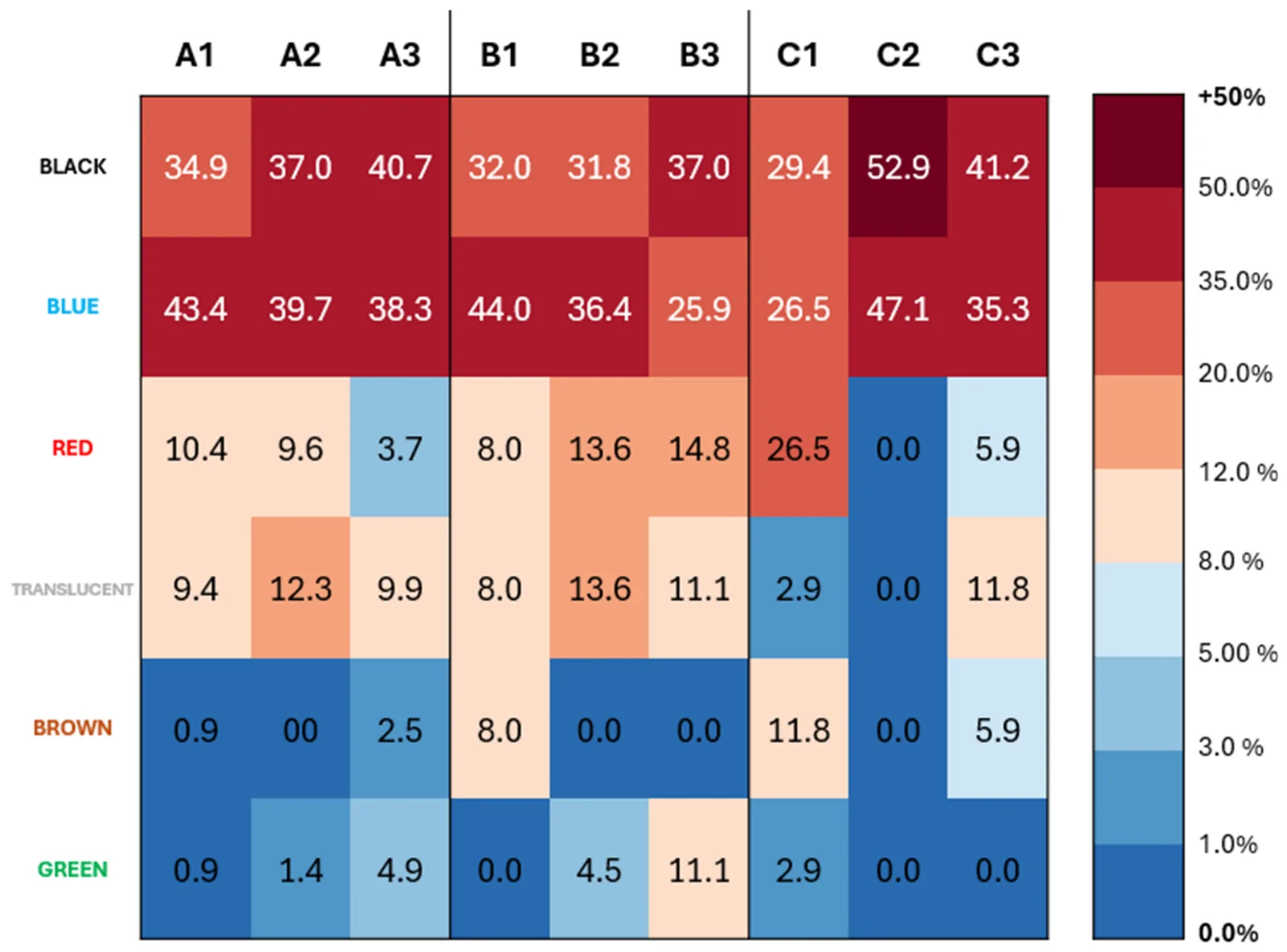
Relative distribution (%) of synthetic particles by colour across different locations and campaigns of sampling.

The colour distribution across the different samples (Figure 2) revealed consistent predominance of dark-colored particles, particularly blue and black, across all sampling locations.

**Figure 3:**
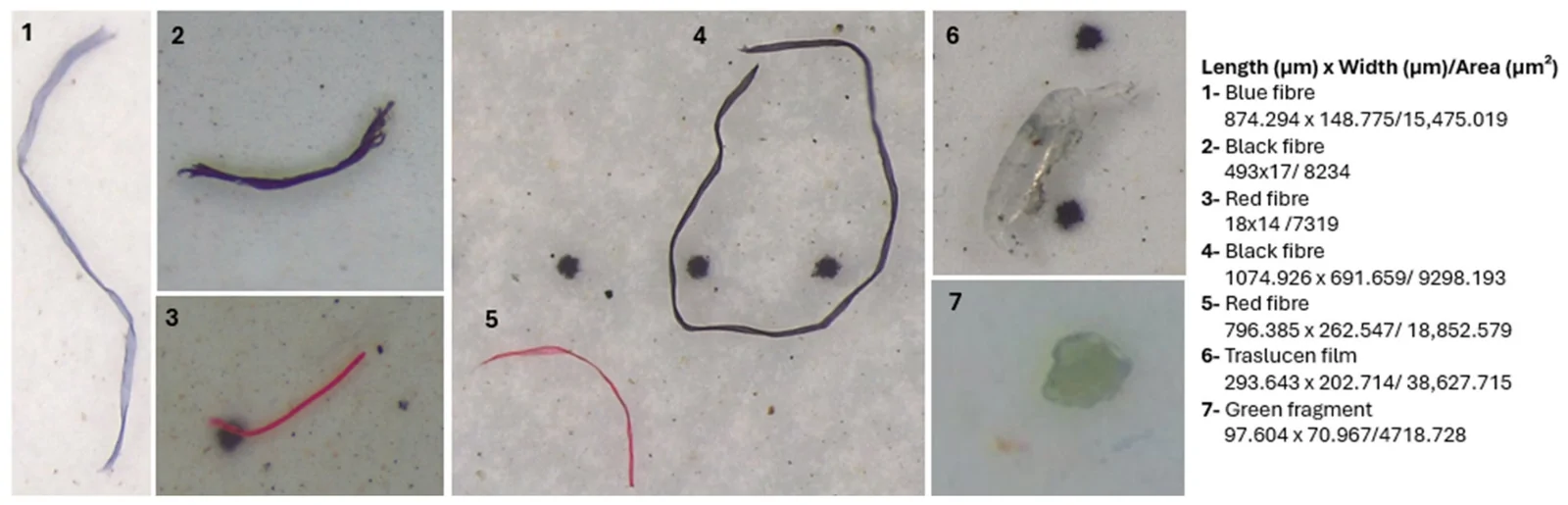
Representative stereomicroscopic images of airborne particles collected in school environments. Particle dimensions are reported as length × width (µm), followed by projected area (µm^2^).

Representative stereomicroscopic images of particles collected in the school environments are presented in Figure 3, complementing the morphological and colour distributions reported above. The examples illustrate fibres of different colours, a translucent film and a green fragment, together with their measured dimensions and projected areas. These optical characteristics describe particle appearance but do not establish their chemical composition or origin; polymer identification was based on µ-FTIR analysis.

### 3.2. Indoor–Outdoor Differences in Particle Abundance and Origin

Regarding the indoor–outdoor comparison, Table 3 summarizes the results obtained from simultaneous indoor–outdoor sampling conducted at School B, including indoor classrooms and the outdoor playground. A higher number of particles was detected indoors than outdoors. Despite this difference in absolute abundance, the relative contribution of natural and synthetic particles was very similar between both environments. Natural particles represented 69.0% of the particles detected indoors and 69.8% outdoors, whereas synthetic particles accounted for 31.0% and 30.2%, respectively. A chi-square test of independence showed no significant association between sampling environment (indoor vs. outdoor) and particle origin (natural vs. synthetic) (χ^2^ (1) = 0.032, *p* = 0.857). Thus, the higher particle abundance observed indoors was not accompanied by a significant change in the relative contribution of natural and synthetic particles.

### 3.3. Polymer Composition and Spatial Distribution

**Figure 4:**
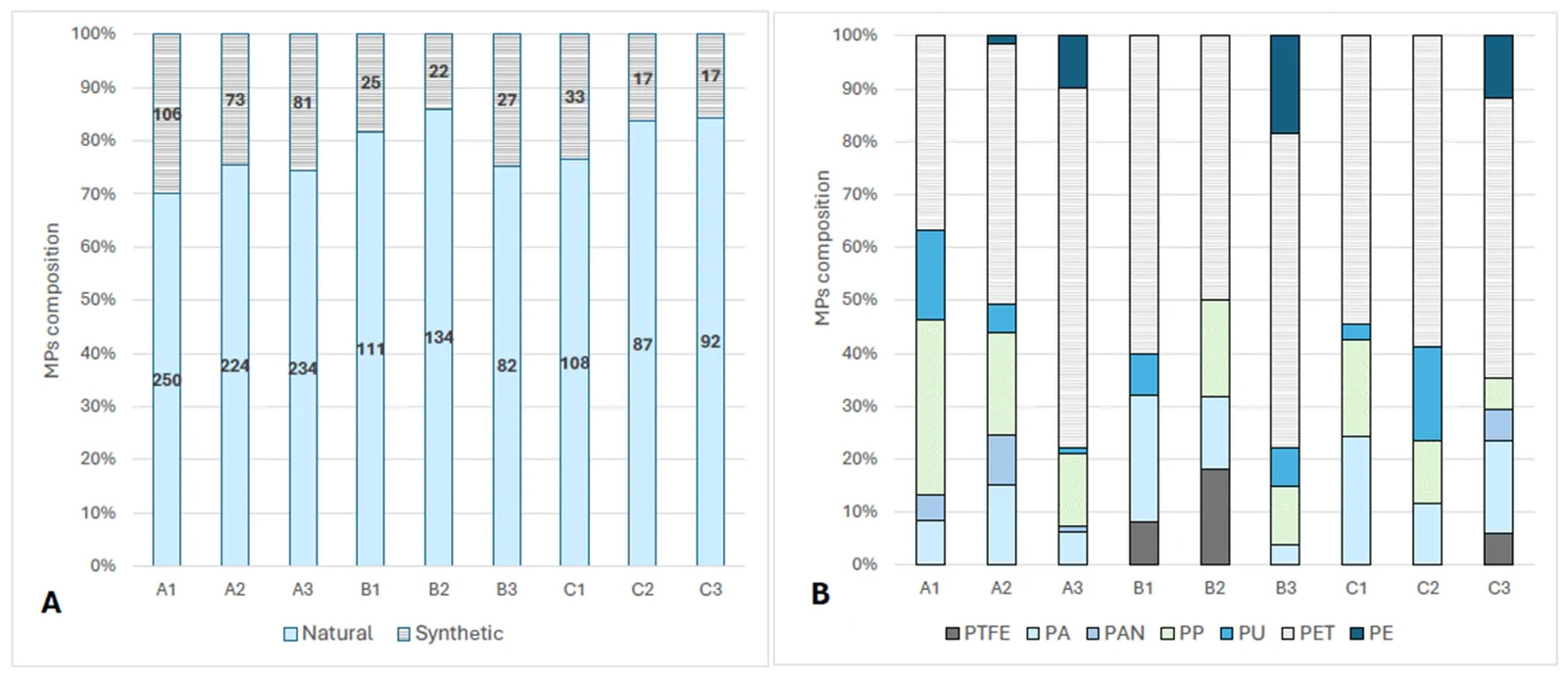
Polymer composition of MPs across sampling locations. (**A**) Relative contribution of natural and synthetic particles. (**B**) Relative distribution of identified synthetic polymers. Values are expressed as percentages relative to the total MPs detected in each location and the absolute particle counts.

Three non-plastic materials were identified and excluded from the synthetic polymer fraction. Among all identified particles, cellulosic materials were the predominant non-plastic component (72.56%), followed by cotton (2.05%) and rayon (0.40%). Overall, particles of natural origin accounted for 70.2–85.9% of the total particles detected at the different sampling sites (Figure 4A).

Seven synthetic polymers were identified: polytetrafluoroethylene (PTFE), polyamide (PA), polyacrylonitrile (PAN), polypropylene (PP), polyurethane (PU), polyethylene terephthalate (PET), and polyethylene (PE). Their overall prevalence followed the order PET >> PP > PA > PU > PE > PAN > PTFE, although their relative contributions varied among sampling locations (Figure 4B).

PET was the predominant synthetic polymer at all three schools and represented 52.12% of the synthetic particles detected across the outdoor sampling campaigns. PP showed a higher relative contribution at School A (21.9%) than at Schools B (9.8%) and C (11.9%). In contrast, PA increased from 9.9% at School A to 13.8% at School B and 17.9% at School C. PU occurred at comparatively similar proportions among the three schools.

Among the less abundant polymers, PAN accounted for 5.2% of the synthetic fraction at School A, was not detected at School B, and represented 2.0% at School C. PTFE was not detected at School A but accounted for 8.7% and 2.0% of the synthetic particles at Schools B and C, respectively. PE occurred at relatively low proportions at all locations, representing 3.8%, 6.2%, and 3.9% at Schools A, B, and C, respectively.

A chi-square test of independence showed a significant association between sampling school and synthetic polymer composition (χ^2^ (12) = 41.77, *p* < 0.0001; Cramér’s V = 0.23), indicating differences in the relative distribution of synthetic polymers among Schools A, B, and C.

**Figure 5:**
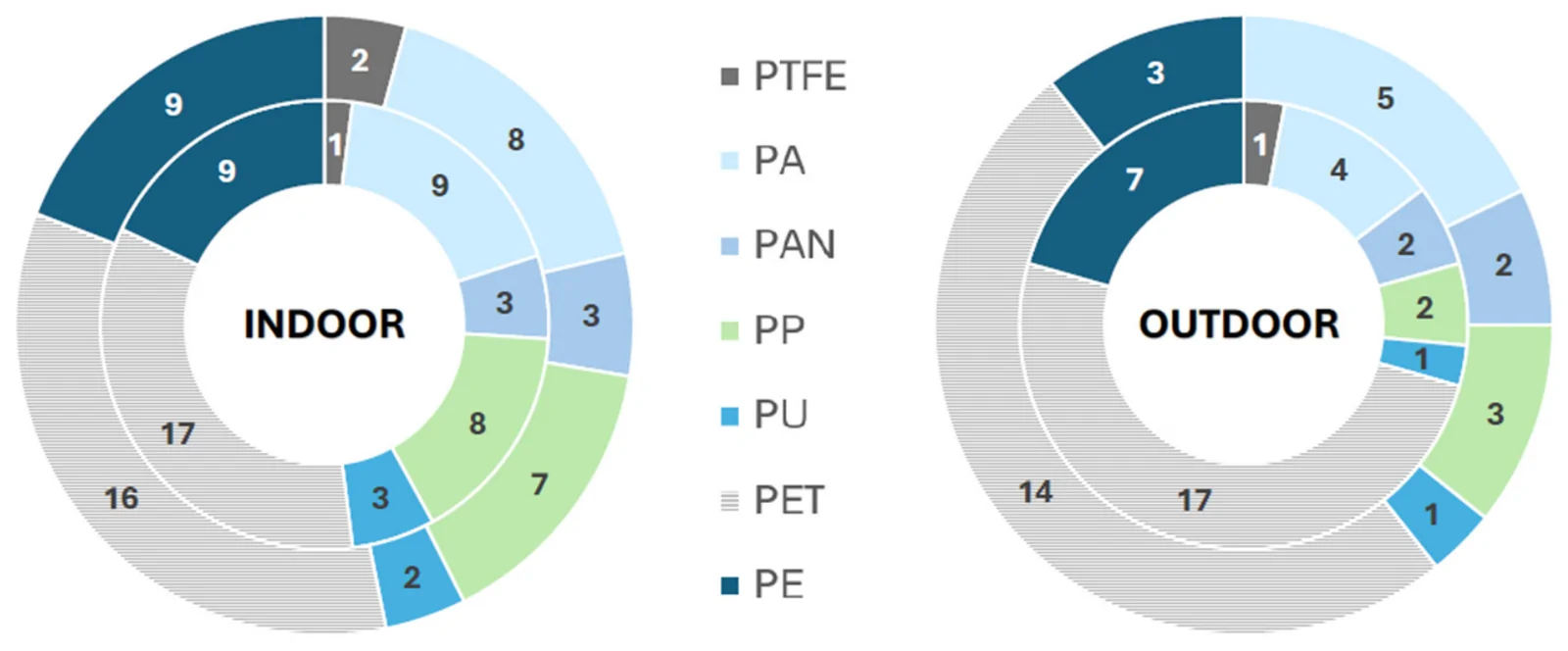
Relative distribution of synthetic polymer types indoors and outdoors. The inner ring represents the first sampling campaign, whereas the outer ring represents the second sampling campaign for each environment.

The indoor–outdoor comparison conducted at School B also showed differences in the relative distribution of synthetic polymers (Figure 5). PP, PA, and PE showed higher relative contributions indoors, whereas PET displayed a greater relative contribution in the outdoor samples.

### 3.4. Assessment of the Aerodynamic Size of Airborne Microplastics

The estimated aerodynamic-size distribution showed that only a small proportion of the collected MPs fell within the finest size categories. Particles with an estimated aerodynamic diameter proxy (d_a_,proxy) < 10 µm represented 0.45% of the total number of particles detected across all samples, whereas particles with d_a_,proxy < 4 µm accounted for 0.13%.

All particles within these aerodynamic-size ranges exhibited a fibrous morphology. Among the outdoor samples, four fibres with d_a_,proxy between 4 and <10 µm were detected at School A, while two fibres were detected at both Schools B and C. Two additional fibres within this range were identified in the indoor samples from School B, whereas none were detected in the simultaneous outdoor samples. For the more restrictive d_a_,proxy < 4 µm category, only three fibres were identified across the complete dataset. No fragments or films were detected within either aerodynamic-size range.

### 3.5. Surface Morphology and Spectral Characteristics of Selected Fibres

**Figure 6:**
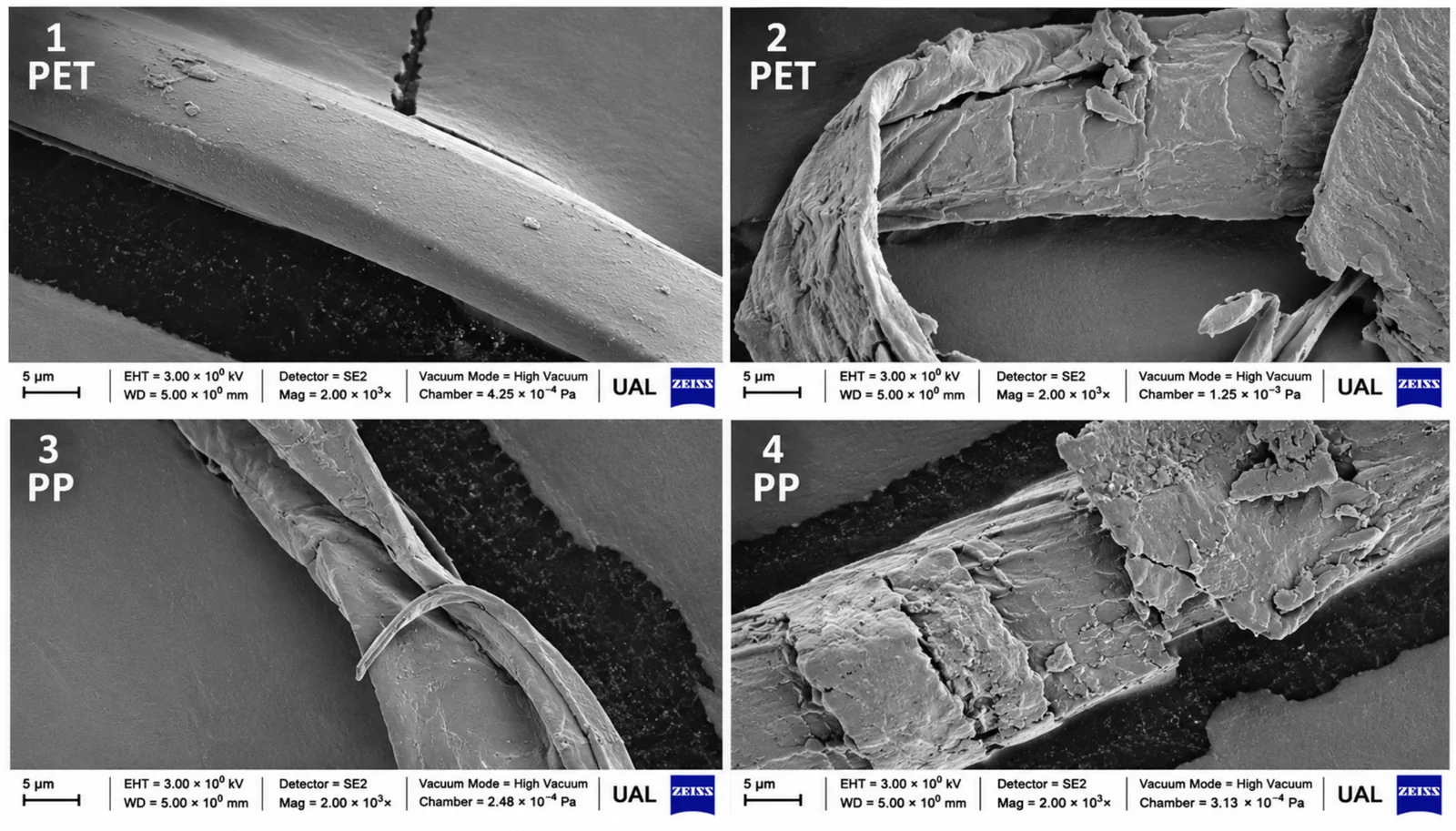
FESEM images of MPs fibres, (**1**) PET particle with 80% spectral match with libraries, (**2**) PET particle with 71% spectral match, (**3**) PP particle with 87% spectral match, (**4**) PP particle with 84% spectral match.

FESEM micrographs revealed clear differences in the surface morphology of the selected PET and PP fibres (Figure 6). Among the PET particles, particle 1 exhibited a comparatively smooth and compact surface with few visible defects, whereas particle 2 showed greater roughness, cavities, fissures, and partial surface delamination. Similar differences were observed for PP. Particle 3 displayed a relatively smooth and compact surface with fewer irregularities, while particle 4 presented greater roughness, cracks, and surface fragmentation.

**Figure 7:**
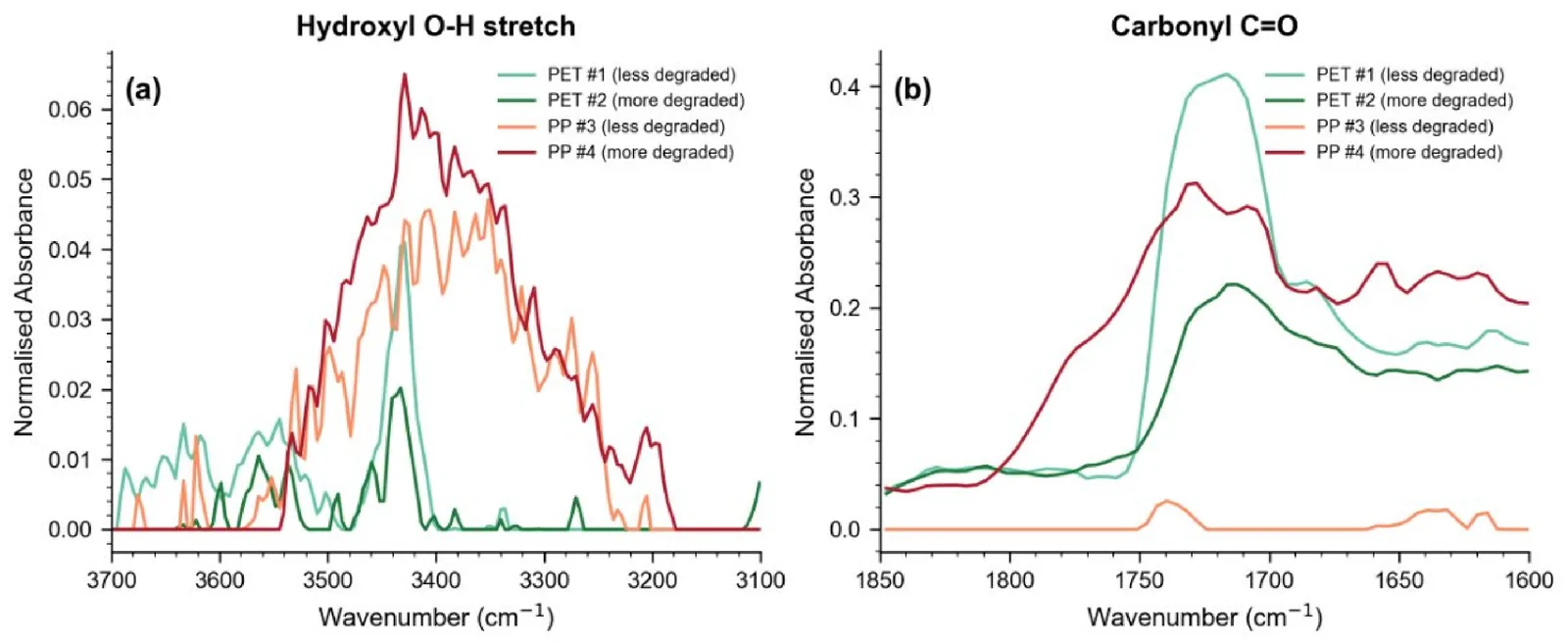
FTIR spectra of PP and PET particles at different stages of degradation. (**a**). Changes in the O–H (~3200–3600 cm^−1^) region; (**b**) changes in the C=O (~1700–1750 cm^−1^) region.

FTIR spectra also showed differences between the less altered and more altered PET and PP particles (Figure 7). Among the PP particles, the more degraded particle exhibited a more pronounced O–H band (~3200–3600 cm^−1^) and an increased signal in the carbonyl region (~1700–1750 cm^−1^) compared with the less degraded particle. For PET, differences were also observed between both particles, although the spectral changes were less pronounced than for PP particles. Variations in the O–H region were moderate, while changes in the carbonyl region were subtle and mainly involved modifications in the intensity and profile of the characteristic ester-related band.

## 4. Discussion

### 4.1. Spatial Variability and Potential Sources of Airborne Particles

The marked predominance of fibres across all sampling locations is consistent with the importance of fibrous materials as a major component of airborne particles in school environments. Their occurrence may be associated with textile-related sources, including clothing, backpacks, upholstery, and other fibrous materials, together with the continuous resuspension of deposited particles caused by human activity.

The substantially higher concentration of synthetic fibres observed at School A compared with Schools B and C suggests that the characteristics of the surrounding environment may influence airborne MPs abundance. School A is in the most densely populated and traffic-influenced setting considered in this study, whereas Schools B and C are located in residential and agricultural peri-urban environments, respectively. Higher traffic intensity, population density, anthropogenic activity, and particle resuspension may therefore contribute to the greater airborne particle load observed at School A.

In contrast to fibres, fragments and films occurred at considerably lower concentrations and showed greater variability among sampling campaigns. The higher abundance of fragments observed at School C during the first campaign may reflect temporally variable or localised particle sources. However, because this increase was not consistently observed across subsequent campaigns, it should be interpreted cautiously rather than attributed to a specific activity or source.

The predominance of particles of natural origin, particularly cellulose, indicates that non-plastic fibrous material represents an important component of airborne particles in school environments. The considerably greater abundance of cellulose compared with cotton and rayon is compatible with the widespread presence and handling of paper, books, cardboard, wooden materials, and cellulose-containing dust in educational settings. These potential sources should nevertheless be considered as plausible interpretations rather than directly demonstrated origins.

The multivariate analysis (Figure 1) further supported the spatial variability observed in the descriptive concentration data. The clear separation of School A from Schools B and C was mainly driven by fibre concentration, consistent with the substantially higher abundance of synthetic fibres measured at this location. The partial overlap between Schools B and C indicates that their morphological profiles were more similar.

Finally, the predominance of blue and black particles observed across the sampling locations may be associated with dyed synthetic textiles and other anthropogenic materials commonly present in urban and school environments, in agreement with previous studies reported by [19].

### 4.2. Indoor–Outdoor Particle Dynamics in School Environments

The higher particle abundance observed indoors may be related to the combined influence of indoor sources, occupant activity, particle resuspension, and reduced dispersion within enclosed environments. Human movement can promote the resuspension of previously deposited particles, while clothing, textiles, furniture, and other materials present in classrooms may contribute additional airborne particles.

However, the very similar proportions of natural and synthetic particles indoors and outdoors indicate that the processes responsible for the higher indoor particle load did not selectively enrich the synthetic fraction. Instead, natural and synthetic particles appear to have been affected in broadly similar proportions. This pattern suggests that indoor accumulation and resuspension may increase the overall particle abundance without substantially altering the relative natural–synthetic composition.

The indoor and outdoor environments should therefore be considered interconnected rather than completely independent compartments. Particle exchange through ventilation, doors, windows, and human movement may contribute to the similarity in particle origin observed between both environments, while lower dispersion and repeated resuspension indoors may explain the greater absolute abundance measured in the classroom.

### 4.3. Variability in Polymer Composition

The marked predominance of cellulosic materials indicates that non-plastic fibres constituted an important fraction of the airborne particles collected in the studied school environments. The presence of coloured cellulosic fibres is compatible with processed anthropogenic materials, since naturally occurring cellulose is less likely to exhibit such colours without previous processing. Their abundance may therefore be related to materials commonly present in schools, including paper, cardboard, books, wooden products, and resuspended cellulose-containing dust.

Among synthetic polymers, the predominance of PET at all three schools may reflect its extensive use in polyester textiles, including clothing, sportswear, upholstery, and other fabrics commonly present in school environments. PET is also widely used in food and beverage packaging, including bottles, containers, and films. Mechanical abrasion and wear of these materials may generate particles that can subsequently become airborne or be resuspended.

Despite the common predominance of PET, the secondary polymer profile differed among the three schools. The higher relative contribution of PP at School A is compatible with the highly urbanised and traffic-influenced characteristics of this location. PP is extensively used in packaging, construction materials, automotive components, non-woven products, and numerous consumer goods. Consequently, road dust, degradation of plastic materials, human activity, and particle resuspension may contribute to its occurrence.

The progressively greater contribution of PA from School A to Schools B and C may be associated with its extensive use in nylon and other synthetic textiles, including clothing, sportswear, backpacks, carpets, and upholstery. The predominantly fibrous morphology observed for PA is consistent with such potential textile-related origins. Similarly, the occurrence of PU may be related to its broad use in foams, coatings, adhesives, furniture, construction materials, and textile products.

PAN showed a more restricted spatial distribution. Because it is extensively used in acrylic fibres, its predominantly fibrous occurrence is compatible with textile-derived emissions. PTFE also showed a heterogeneous distribution and, given its relatively low abundance, its occurrence may reflect localised or intermittent contributions rather than a widespread source. PE was detected at relatively low proportions at all three schools despite its widespread use in packaging, films, and consumer products. However, low airborne abundance should not be interpreted as evidence of limited PE-containing sources, because atmospheric occurrence is also affected by material degradation, particle morphology, resuspension, transport, and sampling efficiency. The occurrence of PE at School C is compatible with a potential contribution from agricultural plastic films, since polyethylene is commonly used in greenhouse coverings. However, PE represented only 3.9% of the synthetic particles at this school, a proportion comparable to School A and lower than School B. Furthermore, PE has numerous non-agricultural applications. Its detection therefore does not establish greenhouse-film degradation as the source of these particles.

The predominance of PET at all three schools indicates a common compositional feature, whereas differences in the relative contributions of secondary polymers, particularly PP and PA, suggest spatial variability in the mixtures of airborne synthetic particles (Figure 4B). These differences may be compatible with variations in local materials and potential emission sources. However, polymer identity alone does not establish particle provenance, and the observed profiles cannot be attributed to a distinct predominant source at each school without additional source-specific evidence.

Differences were also observed between indoor and outdoor polymer profiles at School B. The relatively greater contributions of PP, PA, and PE indoors may be related to the diversity of polymer-containing materials present in classrooms. PP is widely used in school supplies, furniture, storage items, and construction materials, while PA is commonly found in clothing, sportswear, backpacks, and other textile products. PE is also widely used in bags, films, flexible packaging, and other consumer materials. Repeated handling, mechanical wear, occupant activity, and reduced particle dispersion in enclosed environments may facilitate the accumulation and resuspension of these polymers.

Conversely, PET showed a stronger relative predominance outdoors. This should not be interpreted as direct evidence of a larger outdoor PET source, since PET fibres released from textiles may be transported between indoor and outdoor environments through ventilation and human movement. Therefore, the observed indoor–outdoor differences are more likely to reflect a combination of source diversity, material use, resuspension, and particle exchange between both microenvironments rather than completely independent indoor and outdoor sources.

### 4.4. Respiratory Relevance and Methodological Implications of Aerodynamic Size

The very small proportion of particles within the finest estimated aerodynamic-size categories suggests that most MPs collected with the present sampling system exhibited aerodynamic characteristics outside the ranges generally associated with penetration into the deeper respiratory tract. Nevertheless, the detection of fibres with dₐ,proxy below 10 µm 4 µm is relevant from an exposure perspective, since aerodynamic behaviour rather than geometric dimensions alone determines particle transport and deposition within the respiratory tract. These thresholds should therefore be regarded as indicators of respirable-like particle fractions rather than as definitive predictors of respiratory deposition.

An important methodological consideration is that the 25 µm mesh aperture used in the present study does not represent a sharp aerodynamic size cut-off. Aerodynamic diameter is not equivalent to the physical dimensions of an elongated fibre. A fibre may have a small transverse dimension and consequently a low estimated aerodynamic diameter while simultaneously exhibiting a geometric length substantially greater than 25 µm. Such fibres can therefore be retained when they approach the mesh transversely or obliquely, intercept the mesh wires, or bridge across an opening.

This behaviour has previously been demonstrated experimentally for airborne fibres collected using mesh screens. Ref. [23] reported that fibres with aerodynamic diameters in the 1–4 µm range could be efficiently retained on 20 and 30 µm mesh screens, particularly when fibre length exceeded the mesh aperture, identifying interception as an important collection mechanism. This provides a plausible explanation for the recovery of fibres with low dₐ,proxy values despite the nominal 25 µm mesh aperture used in the present study.

However, the recovery of some fibres with small estimated aerodynamic diameters should not be interpreted as evidence that the sampling system captures the entire fine airborne fraction. Particles that do not intercept the mesh structure may pass through its openings, potentially leading to an underestimation of the finest particles. Consequently, the percentages reported here should be interpreted as the proportion of fine particles among those successfully collected rather than as an absolute estimate of their atmospheric abundance.

The low proportion of particles within the finest aerodynamic-size ranges is broadly consistent with previous studies reporting lower abundances of MPs in the smallest airborne fractions. Ref. [24] for example, reported lower microplastic concentrations in PM_2.5_ and PM_1_ than in PM_10_. However, the morphological composition of the finest fraction can vary considerably among studies. Ref. [25] reported a greater contribution of irregular fragments within the 1–5 µm size range, with fibres representing a smaller fraction. Such differences may arise from variation in sampling methodology, analytical size limits, environmental sources, and particle morphology.

Future studies could improve the characterization of the potentially respirable fraction by employing collectors with smaller mesh apertures or alternative size-selective sampling systems. However, decreasing the mesh aperture would increase airflow resistance and pressure requirements, potentially require more powerful pumps and reduce the portability and autonomy of field sampling devices. Future sampler designs should therefore balance fine-particle collection efficiency with airflow requirements, battery autonomy, and field portability.

### 4.5. Polymer-Dependent Degradation Features

The FESEM observations indicate different degrees of surface alteration in the selected PET and PP fibres. In both polymers, the transition from comparatively smooth and compact surfaces to rougher surfaces with cracks, cavities, and fragmentation is consistent with progressive environmental alteration. Because all analysed particles originated from environmental samples, the observed features likely reflect the combined action of physical, chemical, and weathering processes rather than a single degradation pathway.

However, FESEM morphology alone is insufficient to identify the specific mechanisms responsible for polymer degradation. Previous studies have shown that UV exposure and thermal ageing can induce hydroxyl- and carbonyl-containing groups in several polymers, including PP [23,24]. The FTIR results obtained in the present study are consistent with this behaviour, since the more altered PP particle showed a clearer O–H band and a stronger carbonyl signal, both compatible with oxidative degradation.

PET showed a different spectral response. Because PET already contains ester groups, its carbonyl signal is intrinsically strong, and degradation does not necessarily result in a marked increase in carbonyl intensity [25]. Instead, degradation-related changes in PET are more subtle and are more plausibly associated with ester bond cleavage and structural modification than with the formation of large amounts of new oxidised species. This interpretation is supported by the more moderate differences observed in the O–H and carbonyl regions between the two PET particles.

Overall, the combined FESEM and FTIR results highlight the polymer-dependent nature of airborne MP ageing. PP showed clearer spectral evidence of oxidation, whereas PET exhibited more subtle changes associated with its ester-containing structure. These findings support the use of complementary morphological and spectroscopic approaches to evaluate the environmental alteration of airborne microplastic fibres.

## 5. Conclusions

Airborne MPs were detected in all the school environments investigated, confirming their widespread occurrence and revealing clear spatial variability in both particle abundance and synthetic polymer composition. Natural particles, mainly cellulosic, dominated the collected material (70–86% of total particles), while synthetic MPs represented a minor fraction. School A, located in the most densely populated area, showed the highest abundance of airborne particles and synthetic fibres, whereas lower concentrations were observed at Schools B and C. Fibres were the dominant morphology at all locations, supporting the relevance of textile-related emissions. Nevertheless, the contrasting abundance observed among schools indicates that local environmental characteristics and surrounding sources may also influence airborne particle loads. Although PET was the predominant synthetic polymer at all three schools, accounting for 52.12% of the synthetic particles detected in the outdoor campaigns, the relative polymer composition differed significantly among locations. Therefore, the predominance of PET appears to represent a common feature across the studied environments, while the secondary polymer profiles indicate spatial variability that may reflect differences in local material use, urban characteristics, particle resuspension and atmospheric transport.

The indoor–outdoor comparison demonstrated that particle abundance and polymer composition provide complementary information. Particle numbers were higher indoors than outdoors, consistent with particle accumulation, occupant activity, textile shedding and resuspension within the classroom. However, the relative proportions of natural and synthetic particles remained very similar between both environments. In contrast, the synthetic polymer profile showed descriptive differences: PP, PA and PE contributed relatively more indoors, whereas PET showed a stronger relative predominance outdoors. These results suggest that indoor and outdoor environments are interconnected but are influenced by different combinations of sources, material use, resuspension and particle exchange.

Only a very small proportion of the collected particles fell within the finest estimated aerodynamic-size categories. Particles with estimated aerodynamic diameters between 4 and 10 µm represented approximately 0.45% of the total, while those below 4 µm represented 0.13%. All particles in these categories were fibres. These values should be interpreted cautiously because the 25 µm mesh may underestimate the finest airborne fraction and because the estimated aerodynamic diameter represents a semi-quantitative approximation rather than an experimentally determined value. Nevertheless, the results highlight the importance of considering particle morphology, density and aerodynamic behaviour rather than geometric dimensions alone.

Finally, FESEM and FTIR analyses of selected PET and PP fibres revealed different degrees of surface and chemical degradation. The observations indicate that environmental modification is polymer-dependent, with PP showing spectral changes compatible with oxidative processes, whereas PET exhibited more subtle modifications associated with its ester-containing structure.

Overall, the study demonstrates that schools represent dynamic environments for airborne microplastic exposure, in which particle abundance, polymer composition, indoor–outdoor exchange and environmental ageing are jointly influenced by local conditions and human activity.

## Figures and Tables

**Table 1:** Environmental conditions recorded during the airborne sampling campaigns conducted at the three study schools.

*School*	*Sampling*	*Date/Hour*	*Temperature*	*Relative Humidity*
*A*	First sampling	31 October 2025—10:00	12–15 °C	55–70%
	Second sampling	27 November 2025—10:00	5–7 °C	42–52%
	Third sampling	15 December 2025—10:00	4–8 °C	53–60%
*B*	First sampling	20 October 2025—10:00	18–19 °C	71–74%
	Second sampling	23 November 2025—10:00	18–19 °C	49–65%
	Third sampling	18 December 2025—10:00	15–17 °C	43–44%
*C*	First sampling	30 October 2025—10:00	24–25 °C	40–42%
	Second sampling	28 November 2025—10:00	20–22 °C	35–38%
	Third sampling	16 December 2025—10:00	18–20 °C	42–43%
	Indoor–outdoor comparison	13 March 2026—10:00	17–19 °C	70–72%

**Table 2:** Abundance and morphological distribution of collected particles across the three sampling locations, including first, second and third sampling campaigns.

		A			B			C		
		1st sampling	2nd sampling	3rd sampling	1st sampling	2nd sampling	3rd sampling	1st sampling	2nd sampling	3rd sampling
Fibres	Total	342	286	303	134	149	108	122	93	98
	Natural	246	219	226	111	133	81	98	82	85
	Synthetic	96	67	77	23	16	27	24	11	13
Fragments	Total	12	2	7	0	2	1	16	3	8
	Natural	4	2	4	0	0	1	10	2	6
	Synthetic	8	0	3	0	2	0	6	1	2
Films	Total	2	9	5	2	5	0	3	8	3
	Natural	0	3	4	0	1	0	0	3	1
	Synthetic	2	6	1	2	4	0	3	5	2

**Table 3:** Indoor–outdoor comparison of particle abundance at School B.

		Indoor		Outdoor	
		Indoor 1	Indoor 2	Outdoor 1	Outdoor 2
Fibres	Total	137	145	102	89
	Natural	97	107	75	66
	Synthetic	40	38	25	23
Fragments	Total	11	12	7	6
	Natural	2	5	1	1
	Synthetic	9	7	6	5
Films	Total	3	5	3	0
	Natural	2	3	0	0
	Synthetic	1	2	3	0

## Data Availability

The data supporting the findings of this study, including particle counts, polymer identification data, morphometric measurements, and spectroscopic data, are available from the corresponding author upon reasonable request.

## Abbreviations

The following abbreviations are used in this manuscript:

MPs	Microplastics
PET	Polyethylene terephthalate
PP	Polypropylene
PU	Polyurethane
PA	Polyamide
PAN	Polyacrylonitrile
PTFE	Polytetrafluoroethylene
PE	Polyethylene
AED	Aerodynamic diameter
µ-FTIR	Micro-Fourier transform infrared spectroscopy
FESEM	Field emission scanning electron microscopy
ECD	Equivalent circular diameter
PLS-DA	Partial least squares discriminant analysis
LV1/LV2	Latent variable 1/Latent variable 2
PM_1_	Particulate matter ≤ 1 µm
PM_2.5_	Particulate matter ≤ 2.5 µm
PM_10_	Particulate matter ≤ 10 µm
